# Insight into the Skin Mycobiota of *Myotis myotis*: How Age, Sex, and Biometric Traits Correlate with Fungal Diversity

**DOI:** 10.3390/ani15203020

**Published:** 2025-10-17

**Authors:** Justyna Borzęcka, Jakub Suchodolski, Magdalena Cal-Smok, Joanna Furmankiewicz, Rafał Ogórek

**Affiliations:** 1 Department of Mycology and Genetics, Faculty of Biological Sciences, University of Wrocław, Przybyszewskiego Street 63-77, 51-148 Wrocław, Poland; justyna.borzecka@uwr.edu.pl (J.B.); jakub.suchodolski@uwr.edu.pl (J.S.); magdalena.cal@uwr.edu.pl (M.C.-S.); 2 Department of Behavioural Ecology, Faculty of Biological Sciences, University of Wrocław, Sienkiewicza Street 21, 50-335 Wrocław, Poland; joanna.furmankiewicz@uwr.edu.pl

**Keywords:** mycobiota, bats, autumn, cave, Poland, biometrics

## Abstract

**Simple Summary:**

This study reports the fungal diversity on wing and tail membranes of *Myotis myotis*, focusing on age, sex, forearm length, and weight. Overall, we isolated 39 different fungal species, with higher diversity on wing membranes. Males were found to be better fungal reservoirs than females, and the most frequently isolated species was *Apiospora arundinis*, which is an opportunistic human pathogen and plant pathogen. Furthermore, we showed that bats likely increase their pool of fungal species colonizing their body surfaces with age, particularly in males. In females, body mass and forearm length negatively correlated with fungal diversity while in males these relationships were positive.

**Abstract:**

This study examines fungal diversity on the wing and tail membranes of the Greater mouse-eared bat (*Myotis myotis*) in autumn, focusing on age, sex, forearm length, and weight. Samples from 15 bats in the Połom caves (Poland) were cultured at 5 °C, 24 °C, and 37 °C. A total of 39 fungal species were identified, with higher diversity on wing membranes. The highest species count occurred at 24 °C (35 species), followed by 5 °C (19 species), and 37 °C (11 species), indicating most isolates were psychrotolerant or mesophilic. The number of fungal species increased with the number of males (r_S_ = 0.181, *p* = 0.518) and with bat age (r_S_ = 0.190, *p* = 0.497), particularly in males (r_S_ = 0.455, *p* = 0.186), and female age showed a negative correlation (r_S_ = −0.363, *p* = 0.548). In turn, the length of the female forearm as well as their body weight negatively affected the number of species occurring on their wing and tail membranes (r_S_ = −0.667, *p* = 0.219, and r_S_ = −0.975, *p* = 0.004, respectively). In the case of *M. myotis* males, positive effects of forearm length (r_S_ = 0.341, *p* = 0.334) and body weight (r_S_ = 0.210, *p* = 0.559) on the number of fungal species inhabiting them were noted. The most frequently isolated species was *Apiospora arundinis*. The absence of *Pseudogymnoascus destructans* (Pd) suggests caves, rather than bats, are the primary Pd reservoirs. The observed fungal diversity and its correlation with biometric traits may have implications for the health and ecology of *M. myotis*. The study establishes a baseline for understanding fungal-bat interactions, with potential relevance for disease surveillance and conservation strategies.

## 1. Introduction

Bats (Chiroptera) are one of the most widespread groups of mammals, but are increasingly threatened by habitat loss and diseases [1]. The Greater mouse-eared bat (*Myotis myotis*), known for its short-distance migrations between summer roosts and winter hibernation sites, is an exception with a stable population that extends across northeastern, central, and southern Poland and is widely distributed throughout Europe [2,3,4]. The high mobility and seasonal dynamics of these bats influence their microbial diversity and pathogen exposure [5], but the skin mycobiota of this species in autumn remains unexplored. Unlike hibernation, where bats remain in stable microclimates, autumn movement exposes individuals to diverse environmental conditions [6], potentially increasing their contact with new fungal propagules and influencing colonization dynamics.

While microbial diversity in mammals is influenced by host physiology and environmental exposure, the potential role of age and sex in shaping *M. myotis* mycobiota still remains unexplored. Given that microbiota composition shifts with age in other vertebrates and that sex-based differences are often linked to hormonal and immune factors [7], it is plausible that similar patterns might occur in *M. myotis*. Investigating these associations could provide novel insights into host–fungus interactions. Moreover, similar to observations in human populations where changes in skin microbiota and biometric parameters are critical for inducing skin disorders [8], it is hypothesized that a similar link may exist in bats.

Research on the mycobiota of *M. myotis* has predominantly focused on the hibernation period, identifying a spectrum of fungi, including *Pseudogymnoascus destructans* (*Pd*), which causes white-nose syndrome (WNS), a major threat to bats in North America [9]. Recent findings by Ogórek et al. [10] confirmed the absence of *Pd* in *M. myotis* bats during spring emergence from the “Nietoperek” Nature Reserve, although *Pd* was detected in this bat species during winter [11,12,13]. However, comprehensive mycological studies of *M. myotis* in autumn are lacking.

Bats serve as significant reservoirs of fungal propagules within underground ecosystems, with the wing membranes exhibiting the highest fungal diversity and quantity when compared to other body areas [11,14,15]. Despite the anatomical continuity between the bat’s wing and tail membranes, delineated only by the leg bones [16], no studies have yet differentiated the mycobiota between these structures. Such a distinction could yield vital mycological insights.

In light of these considerations, this study aimed to address existing knowledge gaps by examining the diversity of cultivable fungal species of *M. myotis* in autumn—a period largely overlooked in existing research. Additionally, by examining fungal communities on wing and tail membranes alongside age, sex, forearm length, and weight, we aimed to identify novel associations between these studied traits.

## 2. Materials and Methods

### 2.1. Study Area

**Figure 1:**
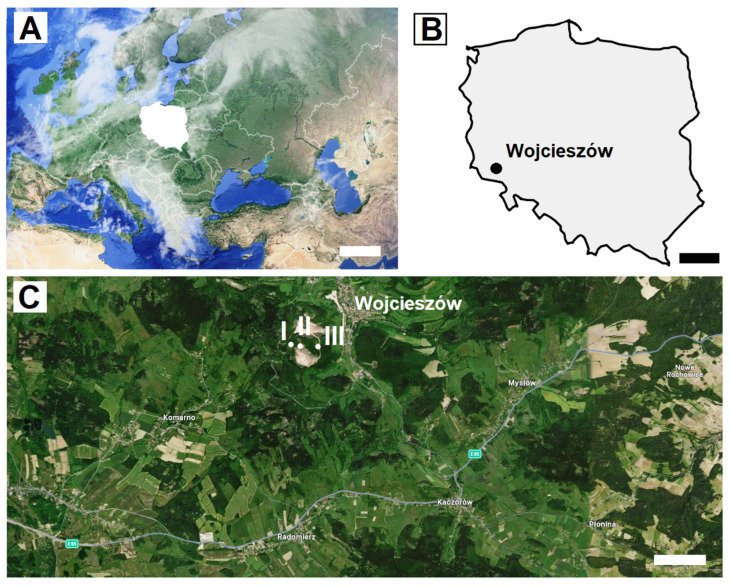
Geographic location of Poland (**A**) and Wojcieszów (**B**), within which the Połom Mt caves, which are located (**C**): I—Północna Duża cave, II—Szczelina Wojcieszowska cave, and III—Nowa cave. Scale bars: (**A**) = 500 km, (**B**) = 100 km, (**C**) = 1 km.

The study has been conducted in three limestone caves (Północna Duża, Nowa, and Szczelina Wojcieszowska) in Połom Mount (667 m a.s.l.) near Wojcieszów in the Western Sudetes in SW Poland (Figure 1). The length of the caves ranges from 103 to 440 m, and the vertical extent from 37 to 112.6 m [17]. Since 2008, the Wojcieszów region has been incorporated into the Special Area of Conservation known as the Kaczawskie Mountains and Kaczawa Foothill (Polish: Góry and Pogórze Kaczawskie) (PLH020037) under the Natura 2000 network, a pan-European system of designated conservation areas [17,18]. Since 2008, the Wojcieszów region has been incorporated into the Special Area of Conservation known as the Kaczawskie Mountains and Kaczawa Foothill (Polish: Góry and Pogórze Kaczawskie) (PLH020037) under the Natura 2000 network, a pan-European system of designated conservation areas [17].

The caves of Połom Mount are locally and regionally important bat hibernacula and autumn swarming sites for fourteen bat species, with the dominance of *M. myotis* (maximally about 360 individuals in winter). The known area from which bats travel to the Połom caves covers at least 120 km. This area includes known wintering sites and autumn swarming locations (up to 120 km away) as well as maternity colonies (up to 18 km away), where bats that winter and swarm in the Połom caves have been recorded [19].

Samples were collected within the Połom caves (Północna Duża cave, Nowa cave and Szczelina Wojcieszowska cave) (Figure 1) between 7 August and 20 October 2021, and involved the determination of biometric characteristics (forearm length, weight, age, and reproductive status) of bats, as well as swabbing from 4 locations on the bats’ bodies (ventral and dorsal side of the wing membranes covering the plagiopatagium, dactylopatagia and propatagium regions, and ventral and dorsal side of tail membrane) for further mycological analyses.

The number of bats captured on each sampling date reflected natural fluctuations in bat activity during the autumn swarming period. The number of bats sampled (n = 15) corresponded to the maximum number permitted by the Regional Directorate for Environmental Protection in Wrocław, ensuring compliance with ethical and conservation guidelines for this protected species.

### 2.2. Sampling Methods

One 6 m long, freshly laundered monofilament mist-net (Ecotone, Poland), mounted on 2.5 m high poles, and one custom-made harp trap (disinfected with alcohol) were used to catch bats during autumn swarming in the Połom caves. Both the net and the harp trap were placed above or in front of the caves.

We determined the species [20], sex, and age, and we measured the weight with a Pesola scale (max. load: 60 g, accuracy: ± 0.3%) and forearm length with a caliper (accuracy: ± 0.1 mm). The age of bats was determined on the basis of external (non-invasive) evaluation of ossification of the epiphyseal joints in the fingers, which are fully ossified in adults and partially ossified with a visible epiphyseal gap in juvenile and subadult individuals during their first summer and autumn [21]. At the end of summer and in autumn, juveniles (born in the same year) pass the juvenile stage and have stronger ossified epiphyseal joints, and are identified as subadults. So, we distinguished between adult and subadult bats.

Swabs were then collected from bats’ wing membranes using sterile swabs saturated with physiological saline solution (0.85% NaCl) and preserved in transport tubes (consisting of a plastic applicator and a 15 cm long viscose swab) according to the protocol of Ogórek et al. [10]. During swab collection, the bats were visually inspected for the presence of fungal infections using a magnifying glass.

Each bat was sampled with four swabs: two for each wing (one for the ventral and one for the dorsal surface), covering the plagiopatagium, dactylopatagia, and propatagium regions, and two for the tail membrane (ventral and dorsal sides). The entire procedure lasted up to 15 min, after which the bats were released at the capture site according to Ogórek et al. [10].

Stringent precautions were taken to prevent cross-contamination, including the use of surgical gowns and changing latex gloves between each sampled bat. The samples were transported in cooling conditions (10 ± 2.0 °C) to the laboratory and stored at 5 ± 0.5 °C until mycological analyses, which were carried out within 7 days [10]. Each swab contained the bat’s identification number, wing/tail membrane type, date of sample collection, sex, and location. In total, we obtained 60 swabs, originating from 15 bats.

### 2.3. Isolation of Fungi from Samples

The isolation of fungi was carried out using conventional phenotypic and culture methods. The swabs from individual membranes were placed in sterile, individually wrapped conical polypropylene test tubes (50 mL) with screw caps (FL Medical, Torreglia, Italy), each containing 3 mL of sterile distilled water. The tubes were then shaken at room temperature (3 min; 500 rpm). Subsequently, the volume of 100 µL and 1000 µL were then spread onto plates containing potato dextrose agar medium (PDA, BioMaxima, Lublin, Poland) in triplicate, and the plates were incubated in darkness at 5 ± 0.5 °C, 24 ± 0.5 °C, and 37 ± 0.5 °C for 5 to 90 days (5 °C corresponding to hibernation site temperature and for psychrophilic and psychrotolerant fungi; 24 °C being the optimal growth temperature for most fungal species; 37 °C representing mammalian body temperature).

After incubation, pure cultures were obtained using the single hyphal tip method [22] and were sub-cultured onto PDA slants (incubated in the dark at 5 or 24 ± 0.5 °C) for molecular identification and further analysis.

### 2.4. Identification of Fungi

A combination of phenotypic and genotypic methods was used for fungal identification. Pure cultures were analyzed with both micro- and macroscopic observations. Preliminary phenotypic identification was performed on PDA and, in the case of some fungi, e.g., *Aspergillus* and *Penicillium*, additionally on Czapek yeast autolysate agar (CYA), Czapek-Dox agar (1.2% agar, BioMaxima, Poland), and malt extract agar (MEA, BioMaxima, Lublin, Poland) [10]. The observed characteristics macromorphology included inter alia colony growth rates and/or growth at various temperatures, texture, degree of sporulation, production of cleistothecia, colors of mycelia, sporulation, soluble pigments, exudates, and colony reverses. In turn, during micromorphological observations, attention was paid to the presence or absence of hyphae, spores and their size, and appearance using preparations from cultures on PDA and/or MEA [22]. The isolates were examined using diagnostic keys and monographs from various sources [23,24,25,26,27,28,29,30].

For species confirmation, the fungal rDNA ITS (internal transcribed spacer) regions were sequenced. DNA was isolated from 20-day-old fungal colonies on PDA medium using the Bead-Beat Micro AX Gravity commercial kit (A&A Biotechnology, Gdańsk, Poland) following the manufacturer’s instructions.

After DNA isolation, verification was performed through electrophoretic separation on a 1.2% agarose gel (1.2 g agarose, 100 mL TBE, 4 µL SimplySafe^TM^) and UV-VIS spectrophotometer (NanoPhotometer^®^ NP80 Implen). Then, fungal rDNA was amplified using the primer pair ITS1 (5′-TCCGTAGGTGAACCTGCGG-3′) and ITS4 (5′-TCCTCCGCTTATTGATATGC-3′) [31]. PCR was conducted in a T100 Thermal Cycler (Bio-Rad, Berkeley, CA, USA), according to Ogórek et al. [32]. The PCR products were verified by electrophoretic separation on a 1.2% agarose gel and a UV-VIS spectrophotometer. Then, PCR products were purified using a Clean-Up kit (A&A Biotechnology) according to the included protocol and sequenced by Macrogen Europe (Amsterdam, The Netherlands) using high-quality Sanger sequencing.

### 2.5. Data Analyses

The BioEdit Sequence Alignment Editor (http://en.bio-soft.net/format/BioEdit.html accessed on 7 December 2024) was utilized to analyze the PCR product sequences. Fungal ITS sequences were compared with those deposited in the GenBank database of the NCBI using the BLAST algorithm (http://www.ncbi.nlm.nih.gov/ accessed on 12 January 2025). Subsequently, the sequences were deposited into GenBank databases. The criteria of Zhang et al. [33] were used to interpret sequences from the GenBank database.

Furthermore, to assess species diversity of fungi on individual bats, the Shannon Diversity Index (H) was employed. This was calculated using the equation: H = −∑ Pi(lnPi), where Pi represents the proportion of each species in the sample [34].

The Spearman’s rank correlation coefficient (r_S_) at α = 0.05 was used to determine the relation between the number of fungal species on the studied bats and their age, sex, forearm length, and weight. For analyses, age was coded as 0 (subadult) or 1 (adult), and sex was coded as 0 (female) or 1 (male).

## 3. Results

Most individuals of *M. myotis* (9 out of 15) were adults. None of the bats had any symptoms of superficial fungal infections. Their body weight ranged from 22.7 g to 36.5 g (mean: 26.5 g), and their forearm lengths ranged from 52.9 mm to 63.4 mm (mean: 60.3 mm) (Table 1).

### 3.1. Fungal Isolation and Identification

Fungal isolation was conducted using conventional phenotypic and culture methods on PDA medium, with incubation at three temperatures (5, 24, and 37 ± 0.5 °C). Classical mycological analysis of the isolates allowed for their classification into 39 groups, which differed in colony macroscopic and/or microscopic morphology. However, some species exhibited only slight morphological differences. Subsequent molecular analyses confirmed the species identity of fungal representatives from each group, verifying the initial phenotypic classifications. In this way, 39 culturable species of fungi were identified, belonging to 3 phyla: Ascomycota (84.62% isolates), Mucormycota (10.25% isolates), and Basidiomycota (5.13% isolates). These fungi represented 3 morphological forms: filamentous fungi (35 species), yeast-like fungi (3 species), and yeast (1 species). The fungal ITS rDNA nucleotide sequences obtained in the study were submitted to GenBank under accession numbers from PV016708 to PV016746. Based on BLAST analysis, all sequences had an E-value of zero, a 100% query cover, and an identity range of 99.18–100%. The sequence lengths ranged from 364 to 570 bp (Table 2).

### 3.2. Fungal Diversity Across Body Regions and the Effect of Age and Sex

**Figure 2:**
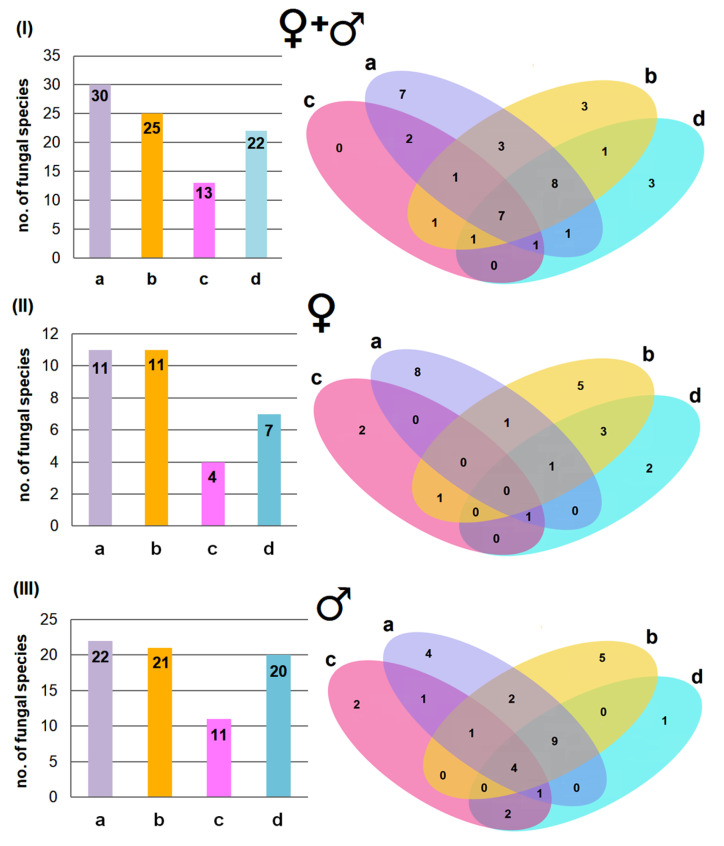
The number of fungal species isolated from the ventral (a) and dorsal (b) side of the wing membranes, and ventral (c) and dorsal (d) side of the tail membrane of *M. myotis*: (**I**) from all studied bats, (**II**) only from females, and (**III**) only from males.

**Figure 3:**
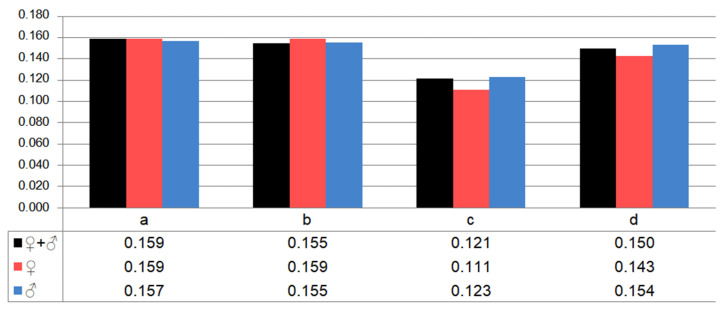
The values of the Shannon Diversity Index (H), calculated for fungal species cultured from the ventral (**a**) and dorsal (**b**) side of the wing membranes, and ventral (**c**) and dorsal (**d**) side of the tail membrane of *M. myotis*.

A total of 39 fungal species were found across all sampled bats, with 30 species found on the ventral side and 25 on the dorsal side of their wing membrane (Figure 2I). Additionally, 13 species were found on the ventral side and 22 on the dorsal side of their tail membrane (Figure 2I). Thus, the wing membranes of *M. myotis* (especially those on the ventral side) harbor a more diverse fungal community than the tail membranes, as also reflected in the Shannon Diversity Index (H) values (Figure 3). Some fungal species were strictly associated with a specific body region, with the highest number found on the ventral side of the wing membranes (7 species), while no species were unique to the ventral side of the tail membranes. Most fungal species, however, were present across multiple body regions (Figure 2I).

The sex of *M. myotis* had some influence on the occurrence of fungal species on the bats’ membranes. Namely, more fungal species were present on the males than on the females, 35 and 24 species, respectively (Figure 2II,III). A similar pattern was also noted for the biodiversity of fungi species inhabiting the individual membranes (Figure 3), as well as the association of fungal species only with a given membrane (Figure 2I–III).

### 3.3. Effect of Incubation Temperature on Fungal Isolation

**Figure 4:**
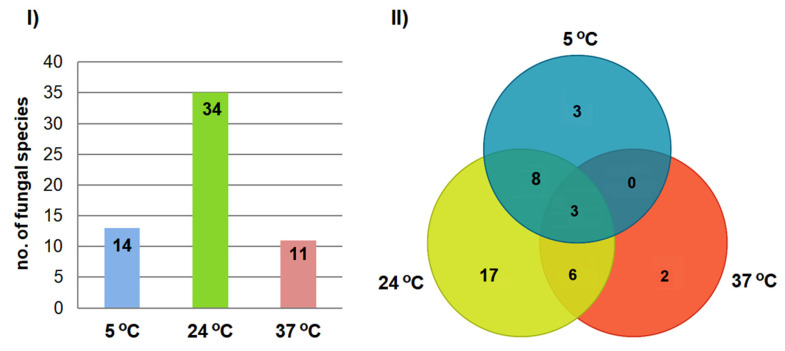
The influence of isolation temperature on the number of cultured fungal species from the wing and tail membranes of *M. myotis*: (**I**) the number of species obtained at a given temperature, and (**II**) the relationships between incubation temperatures and isolated species.

The number of isolated fungal species varied depending on the incubation temperature (Figure 4). The highest species richness was recorded at 24 °C (35 species), while the lowest was at 37 °C (11 species) (Figure 4I). Some species were recovered at multiple temperatures (e.g., 3 out of 39 species were isolated at all three temperatures), whereas others were temperature-specific (e.g., 17 species were only obtained at 24 °C) (Figure 4II).

**Figure 5:**
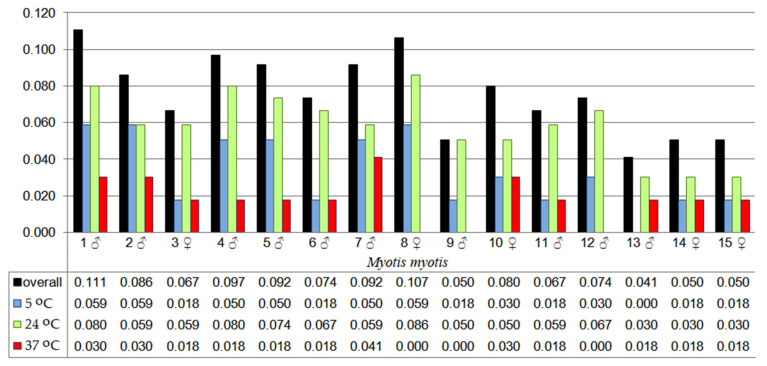
The values of the Shannon Diversity Index (H), calculated for fungal species cultured from the wing and tail membranes of *M. myotis* from particular incubation temperatures, and overall for all temperatures.

Fungal species diversity varied among individual bats and was largely dependent on the incubation temperature, as reflected in the Shannon Diversity Index (H) values. The highest Shannon Index value (0.111) was recorded for *M. myotis* no. 1 (male), while the mean value for all individuals was 0.076. At 5 °C incubation, the highest species diversity (H = 0.059) was observed in three individuals (males no. 1 and no. 2, and female no. 8). At 24 °C, the highest diversity (H = 0.086) was observed in female no. 8, whereas at 37 °C, male no. 7 had the highest diversity value (H = 0.041). The mean diversity values were 0.033 for 5 °C, 0.059 for 24 °C, and 0.018 for 37 °C, with no clear trend observed across temperatures (Figure 5).

### 3.4. Dominant Fungal Species and the Effect of Biometric Features on Fungal Diversity

**Figure 6:**
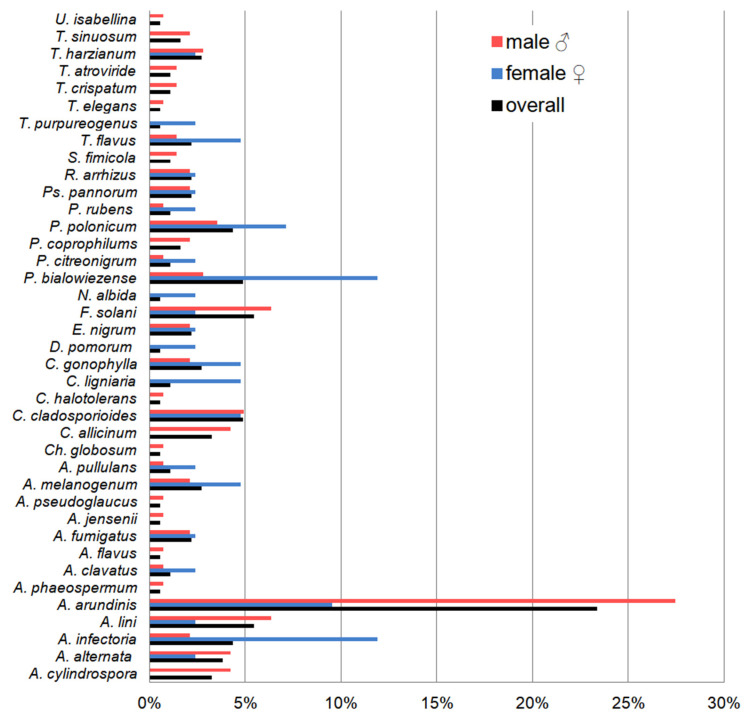
The percentage contribution of each fungal species isolated from the wing and tail membranes of *M. myotis* to the total number of fungi isolated.

*Apiospora arundinis* (filamentous fungi belonging to the Ascomycota phylum) was the most frequently isolated species of the 15 bats studied, regardless of body parts, sex of individuals, and incubation temperature—it constituted 23.3% of all isolated fungal species. In the case of the *M. myotis* sex, the most frequently isolated species was also *A. arundinis* in the case of males, constituting 27.5% of all other species. In turn, in the case of females, two other filamentous species belonging to Ascomycota phylum (*Alternaria infectoria* and *Penicillium bialowiezense*) were obtained the most abundantly, each of them constituting 11.9% of all isolated fungal species (Figure 6, Table A1). *A. arundinis* also dominated in the case of fungi incubated at 5 °C and 24 °C which were cultured from all bats (30.1% and 2.6% of all isolated species, respectively). In turn, the most frequently isolated species at 37 °C was *A. infectoria*, which accounted for 25% of all isolated species. On the other hand, the dominant species on the ventral and dorsal sides of the wing and tail membranes (regardless of the incubation temperatures) was also *A. arundinis*, which accounted for 17.0%, 21.4%, 32.0%, and 27.1% of all obtained species, respectively (Table A1).

The examined biometric features of *M. myotis* (sex, forearm length, weight, and age) had a different effect on the number of fungal species inhabiting the wing and tail membranes of bats. The number of fungi species inhabiting bats (regardless of sex) increased with their age (r_S_ = 0.190, *p* = 0.497) and the increase in the number of males (r_S_ = 0.181, *p* = 0.518). This positive correlation is also maintained when considering only male age *M. myotis* (r_S_ = 0.455, *p* = 0.186), but in the case of female age, this effect is negative (r_S_ = −0.363, *p* = 0.548).

In turn, the length of the bats’ forearm as well as their body weight negatively affected the number of species occurring on the wing and tail membranes of these small mammals both in the overall approach regardless of sex (r_S_ = −0.018, *p* = 0.949 and r_S_ = −0.328, *p* = 0.232, respectively) and in the case of females (r_S_ = −0.667, *p* = 0.219, and r_S_ = −0.975, *p* = 0.004, respectively). In the case of *M. myotis* males, positive effects of forearm length (r_S_ = 0.341, *p* = 0.334) and body weight (r_S_ = 0.210, *p* = 0.559) on the number of fungal species inhabiting them were noted.

## 4. Discussion

This study marks a significant advancement in understanding the seasonal mycobiota dynamics associated with *M. myotis*. The identification of 39 distinct fungal species underscores the high fungal diversity on the membranes of *M. myotis*, with notable differences from previously documented populations. The higher fungal diversity observed in autumn compared to spring populations [10] suggests that bats accumulate fungal propagules throughout their seasonal movements. Wing membranes of bats departing from the caves in spring exhibited only 17 fungal species, while in the present study, 39 species were identified. Interestingly, the common species between the present study and those identified by Ogórek et al. [10] are cosmopolitan species that are often associated with caves, such as *A. fumigatus*, *A. jensenii*, *C. globosum*, and *P. chrysogenum*. However, two species, *P. coprophilum* and *P. citreonigrum*, found in both reports, are rarely associated with underground ecosystems. Therefore, we hypothesize that both species might be common mycoflora of *M. myotis* bats in the studied regions of Poland.

Further supporting seasonal differences in fungal diversity, Borzęcka et al. [15], who researched airborne fungi near hibernation sites of *M. myotis* in “Nietoperek”, reported the occurrence of 32 species, of which *A. fumigatus*, *C. cladosporioides*, *P. bialowiezense*, and *P. chrysogenum* overlap with this study. These species are representative of typical cave fungi. At this point, it can be hypothesized that the diversity of fungal species varies among *M. myotis* bats in the autumn, during hibernation, and in the spring. However, to fully understand the seasonal changes in mycobiota, future research should focus on the fungi typically associated with *M. myotis* membranes during hibernation and summer roosting sites. Such variability in mycobiota may indicate seasonal fluctuations in pathogens, which is critical for developing conservation strategies to protect bat species from fungal diseases. Besides *Pd*, other bat-associated fungal pathogens include *Histoplasma capsulatum*, *Cryptococcus gattii*, *C. neoformans*, and *Paracoccidioides brasiliensis*, which have been reported to cause severe or even fatal infections in humans and other mammals.

It should also be noted that in our study, we used three different incubation temperatures. In culture methods, incubation temperature plays a key role in the number of species isolated from samples [15]. This was confirmed in our study: the highest species biodiversity was observed at 24 °C (35 species), followed by 5 °C (19 species), and 37 °C (11 species), indicating that most isolates were psychrotolerant or mesophilic. Notably, the absence of *Pd* in the present study provides critical insights into the pathogen dynamics within European bat populations. Considering the findings from the “Nietoperek” hibernation site [10,11,12,13], it appears that *M. myotis* bats enter hibernation sites *Pd*-free, co-exist with the pathogen during hibernation, and exit in spring without *Pd* propagules. This cycle suggests that *M. myotis* may not serve as a reservoir for *Pd*, which seems to be sourced from the caves themselves. To confirm this pattern, future research should focus on tracking summer, winter, and spring populations in the Połom caves and comparing them with summer and autumn populations from “Nietoperek.”

In contrast, North American species such as the Little brown bat (*Myotis lucifugus*) and Northern long-eared bats (*Myotis septentrionalis*) exhibit different interactions with *Pd* [5], becoming transiently infected in autumn with infection peaking by late winter. However, the influence of bat mobility and infectiousness on the seasonal timing of pathogen spread to new populations is unknown [5]. We suggest that during summer, despite a high number of contacts and births, bats are not a source of *Pd*, because this fungus species is a typical psychrophile with optimal growth temperatures between 12.5 and 15.8 °C, and an upper critical temperature between 19.0 and 19.8 °C [35]. Although Ballmann et al. [36] demonstrated that *Pd* can still be transmitted in summer by bats as well as by contaminated equipment and clothing, provided that it concerns underground hibernacula. Therefore, it still concerns places that meet the temperature criterion for *Pd* growth.

Understanding the seasonal prevalence of *Pd* in *M. myotis* is crucial, as existing studies suggest that this species tolerates *Pd* infection without significant changes in immune gene regulation, indicating a potential commensal relationship with the pathogen [13,37]. Although *M. myotis* rarely hibernates with other bat species, there have been observed instances of co-hibernation with the Lesser horseshoe bat (*Rhinolophus hipposideros*) [38]. Importantly, subsequent studies have shown that *R. hipposideros* may be adversely affected by *Pd* [39], highlighting the complex interspecies interactions within shared hibernacula and their implications for disease transmission dynamics.

The observed differences in fungal distributions between bat tail and wing membranes can be attributed to various factors. Based on our observations during sample collection, it is often noted that the tail membranes are frequently contaminated with feces and urine, which likely contributes to the unique mycobiota observed in these areas. While there is no specific research discussing this phenomenon directly in bats, it is well-documented that bat guano is a rich source of microorganisms [40], including fungi [41], supporting the idea that contamination from excreta could influence microbial populations on the tail membranes. Additionally, variations in micro-environmental conditions, such as moisture and temperature, alongside the structural differences in skin and sebaceous gland density between the thinner wing and the thicker tail membranes [42], likely influence the occurrence of spores and other fungal propagation structures on the skin of these small mammals. Additionally, the aerodynamics of flight and the generated metabolic heat may affect spore deposition and create distinct microclimates on the wings compared to the tail [16], making the wings more susceptible to a diverse array of fungal species.

Beyond environmental exposure, the foraging behavior of *M. myotis* may contribute to fungal accumulation on wing membranes. This species primarily hunts ground-dwelling carabid beetles (Carabidae) [43], capturing them directly from the floor with its wing membranes. Carabidae carry fungal spores on their exoskeletons and in their gut microbiota [44], suggesting that prey handling facilitates fungal transfer. Given that many of the isolated fungal species, including *A. arundinis*, are plant pathogens, some may originate from beetles previously exposed to vegetation or leaf litter.

Each of the 15 *M. myotis* individuals harbored between 3 to 14 fungal species, with notable variation in fungal diversity across individuals. Our results show that age significantly influenced fungal diversity, with older bats generally hosting a higher number of fungal species. This pattern was particularly evident in males, where a strong positive correlation was observed. Conversely, in females, fungal richness decreased with age. Additionally, forearm length and body weight were negatively correlated with fungal richness, but only in females. These findings suggest that host-related factors, particularly sex and age, may shape fungal colonization patterns in a complex manner.

As this study is the first to examine the relationship between biometric traits and fungal diversity in bats, further research is needed to determine whether these patterns result from immune responses, behavioral factors, or physiological adaptations linked to migration and roosting ecology. Understanding these interactions will be crucial for assessing how host characteristics influence fungal transmission and persistence in bat populations. Future studies involving individually marked bats could provide valuable insights into seasonal and interannual dynamics of skin mycobiota in *Myotis myotis* populations. It is important to recognize that while certain fungal species may establish beneficial relationships with bats, enhancing ecological balance and host health, others display significant risks. Pathogenic fungi, especially within the genus *Aspergillus*, are notable concerns due to their potential to cause invasive infections in immunocompromised hosts [45,46]. Additionally, the production of mycotoxins by species such as *A. fumigatus* [47], *A. clavatus* [48], and *P. chrysogenum* [49] adds complexity to host–pathogen interactions and impacts ecosystem dynamics within bat habitats.

## 5. Conclusions

This study provides the first detailed comparison of fungal species on bat wings and tail membranes in autumn, revealing significant differences in fungal diversity in favor of flight membranes. Specific species associated exclusively with either the wing or tail membranes were identified, suggesting specialized ecological niches within the bat body. Males were found to be better fungal reservoirs than females, although this result may have been influenced by the sample size and the predominance of males. Furthermore, the absence of *Pd* in these bats indicates a potential non-reservoir status for this pathogen in the bat population studied. The most frequently isolated species was *Apiospora arundinis*, which is an opportunistic human pathogen (onychomycosis) and plant pathogen, but no macroscopic symptoms of superficial mycoses were detected in bats. Therefore, it can be assumed that bats are immune to this opportunistic pathogen and may only be its reservoir. We also confirmed the effectiveness of using different incubation temperatures to obtain the widest possible species spectrum during fungal isolation from environmental samples. Furthermore, we showed that bats likely increase their pool of fungal species colonizing their body surfaces with age. Therefore, we strongly believe that this study provides a foundation for future efforts to understand the biology and ecology of *M. myotis* bats in their natural habitats in terms of the acquisition of mycobiota.

## Figures and Tables

**Table 1:** Biometric characteristics of *M. myotis* bats (10 males and 5 females; ♂ and ♀, respectively) collected from 3 caves in the Połom Mt area: “^1^–“—no data. None of the individuals displayed superficial mycoses.

Bat		Sampling			Sex	Forearm Length (mm)	Weight (g)	Age
No.	Ring No.	Location	Date	Hour				
1	C20744PL	Szczelina Wojcieszowska cave	07.09.2021	2:30	male	61.3	25.0	adult
2	C20743PL		07.09.2021	1:50	male	55.0	24.5	subadult
3	C20782PL		07.09.2021	22:45	female	61.4	29.5	adult
4	C20745PL		07.09.2021	1:50	male	62.0	25.0	adult
5	C20741PL		07.09.2021	2:30	male	61.2	25.0	subadult
6	C20742PL		07.09.2021	2:30	male	61.8	23.0	subadult
7	C20781PL		15.09.2021	22:15	male	52.9	26.5	adult
8	–^1^	Nowa cave	07.09.2021	23:00	female	61.5	23.0	adult
9	–		07.09.2021	00:20	male	61.0	24.5	subadult
10	–		07.09.2021	23:30	female	61.1	26.0	subadult
11	C20710PL	Północna Duża cave	07.09.2021	22:50	male	60.9	22.7	adult
12	C20929PL		07.09.2021	1:10	male	60.1	29.5	adult
13	–		29.09.2021	21:30	male	58.2	25.5	subadult
14	–		29.09.2021	20:55	female	63.0	36.5	adult
15	C20953PL		20.10.2021	19:50	female	63.4	32.0	adult

**Table 2:** Results of the BLAST analyses of fungi cultured from the wing and tail membranes of *M. myotis* bats. All E values were zero, and all Query Cover values were 100%. Isolates UWR_438, UWR_467, UWR_471, and UWR_476 belong to the Mucormycota phylum, UWR_456 and UWR_460 belong to Basidiomycota, and others belong to Ascomycota.

Isolate Number	Identified Fungi	GenBank Accession No.	The Sequence Length (bp)	Identity (%)	Accession
UWR_438	*Absidia cylindrospora*	PV016708	513	99.81	MN817778.1
UWR_439	*Alternaria alternata*	PV016709	518	100	MN907440.1
UWR_440	*Alternaria infectoria*	PV016710	522	100	MN534845.1
UWR_441	*Alternaria lini*	PV016711	517	100	OL687532.1
UWR_442	*Apiospora arundinis*	PV016712	506	100	KF144885.1
UWR_443	*Arthrinium phaeospermum*	PV016713	413	100	OW984648.1
UWR_444	*Aspergillus clavatus*	PV016714	488	100	MK271292.1
UWR_445	*Aspergillus flavus*	PV016715	533	100	MT447477.1
UWR_446	*Aspergillus fumigatus*	PV016716	527	100	MT558940.1
UWR_447	*Aspergillus jensenii*	PV016717	507	100	MT582748.1
UWR_448	*Aspergillus pseudoglaucus*	PV016718	482	100	MT582752.1
UWR_449	*Aureobasidium melanogenum*	PV016719	516	100	MH855849.1
UWR_450	*Aureobasidium pullulans*	PV016720	514	100	MT035961.1
UWR_451	*Chaetomium globosum*	PV016721	473	100	MN654349.1
UWR_452	*Cladosporium allicinum*	PV016722	463	100	OK445643.1
UWR_453	*Cladosporium cladosporioides*	PV016723	493	100	MT781987.1
UWR_454	*Cladosporium halotolerans*	PV016724	486	100	MN826823.1
UWR_455	*Coniochaeta ligniaria*	PV016725	461	100	MT920581.1
UWR_456	*Coprinopsis gonophylla*	PV016726	570	100	MW560230.1
UWR_457	*Didymella pomorum*	PV016727	466	100	KU554583.1
UWR_458	*Epicoccum nigrum*	PV016728	495	100	KP794171.1
UWR_459	*Fusarium solani*	PV016729	430	100	OP482353.1
UWR_460	*Naganishia albida*	PV016730	525	100	OM021981.1
UWR_461	*Penicillium bialowiezense*	PV016731	500	100	MT582764.1
UWR_462	*Penicillium citreonigrum*	PV016732	364	99.18	EF198645.1
UWR_463	*Penicillium coprophilum*	PV016733	484	100	MT410465.1
UWR_464	*Penicillium polonicum*	PV016734	509	100	MT582786.1
UWR_465	*Penicillium rubens*	PV016735	476	100	MT079294.1
UWR_466	*Pseudogymnoascus pannorum*	PV016736	459	99.78	MW019476.1
UWR_467	*Rhizopus arrhizus*	PV016737	544	100	MT590596.1
UWR_468	*Sordaria fimicola*	PV016738	510	100	MN341414.1
UWR_469	*Talaromyces flavus*	PV016739	437	100	MT074667.1
UWR_470	*Talaromyces purpureogenus*	PV016740	509	100	MN206956.1
UWR_471	*Thamnidium elegans*	PV016741	561	100	JN206059.1
UWR_472	*Trichocladium crispatum*	PV016742	495	100	OP699917.1
UWR_473	*Trichoderma atroviride*	PV016743	532	100	OP539101.1
UWR_474	*Trichoderma harzianum*	PV016744	394	100	MT584872.1
UWR_475	*Trichoderma sinuosum*	PV016745	374	99.73	JQ272463.1
UWR_476	*Umbelopsis isabellina*	PV016746	548	100	MZ078794.1

## Data Availability

Data will be made available upon request.

## Appendix A

**Table A1:** Diversity of fungal species cultured from the ventral (a) and dorsal (b) side of the wing membranes (the plagiopatagium, dactylopatagia, and propatagium regions), and ventral (c) and dorsal (d) sides of the tail membrane of *M. myotis* using incubation at different temperatures (5, 24 and 37 ± 0.5 °C): “–”—no detected.

Fungal Species	*Myotis myotis*														
	1 ♂	2 ♂	3 ♀	4 ♂	5 ♂	6 ♂	7 ♂	8 ♀	9 ♂	10 ♀	11 ♂	12 ♂	13 ♂	14 ♀	15 ♀
*Absidia cylindrospora*	–	–	–	–	24 °C ^(c, d)^	24 °C ^(a, b, c, d)^	–	–	–	–	–^1^	–	–	–	–
*Alternaria alternata*	5 °C ^(a)^	–	–	–	–	–	24 °C ^(a, b, d)^; 37 °C ^(a, b)^	5 °C ^(b)^	–	–	–	–	–	–	–
*Alternaria infectoria*	–	–	37 °C ^(a)^	–	24 °C ^(d)^	–	37 °C ^(d)^	24 °C ^(b)^	–	37 °C ^(a)^	24 °C ^(c)^	–	–	–	37 °C ^(b, d)^
*Alternaria lini*	24 °C ^(b, d)^	–	–	–	24 °C ^(d)^	24 °C ^(d)^	24 °C ^(a, b, d)^	–	24 °C ^(a)^	–	–	24 °C ^(b)^	–	–	24 °C ^(a)^
*Apiospora arundinis*	5 °C ^(b)^; 24 °C ^(a, c, d)^	5 °C ^(b)^; 24 °C ^(a, d)^	5 °C ^(b)^; 24 °C ^(b)^	5 °C ^(a, b, d)^; 24 °C ^(a, b, d)^	5 °C ^(b)^; 24 °C ^(d)^	5 °C ^(b)^; 24 °C ^(a, b, c)^	5 °C ^(a, d)^; 24 °C ^(a, d)^	24 °C ^(c, d)^	5 °C ^(a, b, c, d)^; 24 °C ^(b, c, d)^	–	–	5 °C ^(a, c, d)^; 24 °C ^(a, b, c, d)^	24 °C ^(c, d)^	–	–
*Arthrinium phaeospermum*	–	–	–	–	–	–	24 °C ^(d)^	–	–	–	–	–	–	–	–
*Aspergillus clavatus*	5 °C ^(c)^	–	24 °C ^(a)^	–	–	–	–	–	–	–	–	–	–	–	–
*Aspergillus flavus*	–	–	–	–	–	–	–	–	–	–	–	–	37 °C ^(b)^	–	–
*Aspergillus fumigatus*	24 °C ^(b)^	–	–	–	–	–	–	24 °C ^(c)^	–	–	24 °C ^(a)^; 37 °C ^(d)^	–	–	–	–
*Aspergillus jensenii*	24 °C ^(d)^	–	–	–	–	–	–	–	–	–	–	–	–	–	–
*Aspergillus pseudoglaucus*	24 °C ^(d)^	–	–	–	–	–	–	–	–	–	–	–	–	–	–
*Aureobasidium melanogenum*	–	37 °C ^(d)^	24 °C ^(d)^	24 °C ^(a, d)^	–	37 °C ^(d)^	–	–	–	–	–	–	–	37 °C ^(d)^	–
*Aureobasidium pullulans*	–	–	–	–	–	–	–	5 °C ^(a)^	–	–	24 °C ^(b)^	–	–	–	–
*Chaetomium globosum*	37 °C ^(a)^	–	–	–	–	–	–	–	–	–	–	–	–	–	–
*Cladosporium allicinum*	5 °C ^(b)^	5 °C ^(d)^	–	5 °C ^(a, d)^	5 °C ^(b)^	–	5 °C ^(b)^	–	–	–	–	–	–	–	–
*Cladosporium cladosporioides*	–	5 °C ^(b, d)^	–	5 °C ^(b, d)^	–	–	5 °C ^(a, b, d)^	–	–	5 °C ^(b, d)^	–	–	–	–	–
*Cladosporium halotolerans*	–	–	–	–	–	–	–	–	–	–	24 °C ^(b)^	–	–	–	–
*Coniochaeta ligniaria*	–	–	–	–	–	–	–	24 °C ^(a)^	–	–	–	–	–	5 °C ^(a)^	–
*Coprinopsis gonophylla*	24 °C ^(c)^	–	–	–	24 °C ^(c)^	–	24 °C ^(b, c)^	24 °C ^(d)^	–	24 °C ^(b)^	–	–	–	–	–
*Didymella pomorum*	–	–	24 °C ^(a)^	–	–	–	–	–	–	–	–	–	–	–	–
*Epicoccum nigrum*	–	–	–	–	24 °C ^(b)^	–	5 °C ^(a, b)^	5 °C ^(a)^	–	–	–	5 °C ^(a)^	–	–	–
*Fusarium solani*	24 °C ^(a, b)^	24 °C ^(d)^	–	24 °C ^(a, c)^; 37 °C ^(b)^	–	24 °C ^(d)^	–	–	–	24 °C ^(b)^	24 °C ^(a, b)^	–	–	–	–
*Naganishia albida*	–	–	–	–	–	–	–	–	–	–	–	–	–	–	24 °C ^(a)^
*Penicillium bialowiezense*	–	5 °C ^(b)^; 24 °C ^(c)^	–	24 °C ^(a)^	–	24 °C ^(d)^	–	5 °C ^(a)^; 24 °C ^(a, c)^	24 °C ^(d)^	–	–	–	–	24 °C ^(c, d)^	–
*Penicillium citreonigrum*	24 °C ^(b)^	–	–	–	–	–	–	24 °C ^(d)^	–	–	–	–	–	–	–
*Penicillium coprophilums*	–	–	–	24 °C ^(a)^	–	24 °C ^(a)^	–	–	–	–	–	24 °C ^(a)^	–	–	–
*Penicillium polonicum*	–	5 °C ^(c)^; 24 °C ^(b)^	–	–	5 °C ^(b)^; 37 °C ^(a)^	–	37 °C ^(b)^	5 °C ^(c)^	–	5 °C ^(b)^	–	–	–	–	5 °C ^(c)^
*Penicillium rubens*	5 °C ^(a)^	–	–	–	–	–	–	–	–	24 °C ^(b)^	–	–	–	–	–
*Pseudogymnoascus pannorum*	–	–	–	–	–	–	–	24 °C ^(b)^	–	–	5 °C ^(a, b, d)^	–	–	–	–
*Rhizopus arrhizus*	–	37 °C ^(b)^	24 °C ^(b)^	5 ° C ^(d)^	–	–	–	–	24 °C ^(a)^	–	–	–	–	–	–
*Sordaria fimicola*	–	–	–		–	–	–	–	–	–	–	24 °C ^(a, b)^	–	–	–
*Talaromyces flavus*	–	–	–	24 °C ^(c, d)^	–	–	–	–	–	37 °C ^(a)^	–	–	–	24 °C ^(b)^	–
*Talaromyces purpureogenus*	–	–	–	–	–	–	–	–	–	24 °C ^(a)^	–	–	–	–	–
*Thamnidium elegans*	–	–	–	–	5 °C ^(a)^	–	–	–	–	–	–	–	–	–	–
*Trichocladium crispatum*	–	–	–	24 °C ^(c)^	–	–	–	–	–	–	–	–	24 °C ^(a)^	–	–
*Trichoderma atroviride*	–	–	–	–	24 °C ^(c, d)^	–	–	–	–	–	–	24 °C ^(a)^	–	–	–
*Trichoderma harzianum*	37 °C ^(b, d)^	24 °C ^(a, d)^	–	–	–	–	–	24 °C ^(d)^	–	–	–	–	–	–	–
*Trichoderma sinuosum*	–	–	–	–	–	–	–	–	–	–	–	24 °C ^(a, b, d)^	–	–	–
*Umbelopsis isabellina*	–	–	–	24 °C ^(b)^	–	–	–	–	–	–	–	–	–	–	–
